# Scaffolds Based on Poly(3-Hydroxybutyrate) and Its Copolymers for Bone Tissue Engineering (Review)

**DOI:** 10.17691/stm2022.14.5.07

**Published:** 2022-09-29

**Authors:** A.P. Bonartsev, V.V. Voinova, A.V. Volkov, A.A. Muraev, E.M. Boyko, A.A. Venediktov, N.N. Didenko, A.A. Dolgalev

**Affiliations:** Associate Professor, Department of Bioengineering, Faculty of Biology; Lomonosov Moscow State University, 1–12 Leninskiye Gory, Moscow, 119234, Russia;; Senior Researcher, Department of Biochemistry, Faculty of Biology; Lomonosov Moscow State University, 1–12 Leninskiye Gory, Moscow, 119234, Russia;; Senior Researcher; N.N. Priorov National Medical Research Center of Traumatology and Orthopedics, 10 Priorova St., Moscow, 127299, Russia; Associate Professor, Department of Pathological Anatomy, Medical Institute; Peoples’ Friendship University of Russia, 6 Miklukho-Maklaya St., Moscow, 117198, Russia;; Professor, Department of Maxillofacial Surgery and Surgical Dentistry; Peoples’ Friendship University of Russia, 6 Miklukho-Maklaya St., Moscow, 117198, Russia;; Teacher, Essentuki Branch; Stavropol State Medical University, 310 Mira St., Stavropol, 355017, Russia;; Director; Cardioplant LLC, 1B Tsentralnaya St., Bldg. 2, Penza, 440004, Russia; Assistant, Department of Pathological Physiology; Stavropol State Medical University, 310 Mira St., Stavropol, 355017, Russia;; Associate Professor, Professor, Department of General and Pediatric Dentistry; Stavropol State Medical University, 310 Mira St., Stavropol, 355017, Russia; Head of the Center for Innovation and Technology Transfer of the Research and Innovation Association; Stavropol State Medical University, 310 Mira St., Stavropol, 355017, Russia;

**Keywords:** scaffolds, matrices, polyhydroxyalkanoates, poly(3-hydroxybutyrate), mesenchymal stem cells, bone defects, osteoinductive properties, bone regeneration

## Abstract

Biodegradable and biocompatible polymers are actively used in tissue engineering to manufacture scaffolds. Biomedical properties of polymer scaffolds depend on the physical and chemical characteristics and biodegradation kinetics of the polymer material, 3D microstructure and topography of the scaffold surface, as well as availability of minerals, medicinal agents, and growth factors loaded into the scaffold. However, in addition to the above, the intrinsic biological activity of the polymer and its biodegradation products can also become evident. This review provides studies demonstrating that scaffolds made of poly(3-hydroxybutyrate) (PHB) and its copolymers have their own biological activity, and namely, osteoinductive properties. PHB can induce differentiation of mesenchymal stem cells in the osteogenic direction *in vitro* and stimulates bone tissue regeneration during the simulation of critical and non-critical bone defects *in vivo*.

## Introduction

Tissue engineering is a multidisciplinary scientific and technological field of study that combines the latest achievements in engineering, material science, cell biology, biochemistry and medicine, and provides new approaches to restore the functions of human tissues and body organs. This is a fundamentally different treatment paradigm compared to surgery and transplantation. It is based not on tissue replacement or functional compensation approaches, but rather on regeneration, when the body can independently restore damaged tissue under appropriate conditions.

There are some clinical conditions such as trauma, skeletal abnormalities, infection, osteoporosis, tumor resection, chronic inflammatory injury, and necrosis that result in bone defects. At present, the “gold standard” of treatment is autologous bone grafting to the major extent. However, this traditional method has significant disadvantages, such as limited availability of transplanted biomaterial and intense pain at the donor site. Bone tissue engineering may be a promising alternative to overcome these problems, it involves the joint use of cells (primarily mesenchymal stem cells, MSCs), scaffolds, and bioactive molecules. Their combination will provide for intercell communication and the necessary interaction between cells and biomaterial, which will facilitate the best therapeutic effect [1-3].

Scaffolds are required as a temporary substrate for cell attachment and growth, as well as for accumulation of extracell matrix. The best simulation of the bone tissue by scaffolds used in tissue engineering is achieved if the scaffolds have a three-dimensional (3D) microstructure with high porosity and defined pore size, the required topography, biocompatibility, and acceptable mechanical properties. It is necessary to ensure circulation of the intercell fluid, directed attachment, migration, proliferation, and differentiation of cells, as well as their optimal integration with the neighboring tissue. Various types of biomaterials can be used to create such scaffolds: metals, ceramics, synthetic or natural polymers. Biodegradable and biocompatible polymers of natural or synthetic origin are preferrable as scaffolds for bone tissue regeneration.

There are different methods to manufacture scaffolds with the desired microstructure: electrospinning, leaching, foaming, particle aggregation, sublimation drying, thermally induced phase separation, microcasting, microfiber spinning, and rapid prototyping (including 3D printing) [3-6]. 3D printing is one of the most intensively developing methods, as it allows to obtain the scaffolds with the required shape, microstructure, and physical and chemical properties. However, not all materials, especially biomaterials of natural origin (in pure form, not in the form of composites with polymers), such as polyhydroxyalkanoates (PHA), obtained through bacterial biosynthesis (primarily, poly(3-hydroxybutyrate) (PHB) homopolymer and its copolymers with other 3-hydroxycarboxylic acids); specific ceramic and silicate biomaterials (zirconium dioxide, bioglass (e.g., 45S5 grade), montmorillanite, saponite, hydroxyapatite, β-tricalcium phosphate); allomaterials and xenomaterials obtained by processing animal tissues, cadaveric and biopsy material are suitable for processing in devices for direct rapid 3D prototyping. The main physical method of processing materials therein is melting, caking, and photocuring, which is either not applicable, or can significantly change (spoil) physical and chemical and biological properties of the mentioned materials [3, 7].

The method of electrospinning is also being actively developed to derive fibrous scaffolds from various polymers and their composites, including nanofiber scaffolds. The main advantages of this method include the following: the resulting scaffolds have a biomimetic structure similar to the fibrous structure of the skin, soft connective tissue, mucous membrane, and muscle tissue. The fibers are formed from a solution, emulsion or dispersion of polymers and their composites, which is a great deal softer processing method compared to melting and caking. Finally, this method ensures manufacturing of scaffolds with a pre-set composition, microstructure, and physical and chemical properties [3, 8].

Mesenchymal stem cells are currently being actively introduced into medical practice as a key active component of new cell technologies and tissue engineering as these cells can be grown in the needed volume and then controlled at the start of their differentiation in the required direction, and namely, osteogenic, chondrogenic, myogenic, neurogenic, etc. MSCs can be clinically applied as a promising cell therapeutic agent. Unlike specialized cells (fibroblasts, osteoblasts, and chondroblasts), the use of MSCs ensures replacement of small cells of endogenous origin in degenerative or regenerative processes and renewal of cells with a high proliferative potential. Moreover, MSC use results in a more adequate response of the immune system after their transplantation *in vivo*. Studies in various animal models demonstrated that MSCs can be used to restore or regenerate damaged bones, cartilages, skin, or myocardial tissues [9-11].

## Scaffolds from poly(3-hydroxybutyrate) and its copolymers for bone tissue engineering: studies on cell cultures *in vitro*

Biodegradable and biocompatible polymers of natural or synthetic origin, PHA, are considered one of the most promising biomaterials to form scaffolds for bone tissue regeneration [12, 13]. PHA can be divided into two groups in line with their chemical structure and method of obtaining: 1) poly(2-hydroxyalkanoates) obtained by chemical synthesis (such as polylactide — PLA, polyglycolide — PGA, and their copolymers — PGLA, poly-ε-caprolactone — PGL); 2) poly(3-hydroxyalkanoates) obtained by bacterial biosynthesis (such as PHB and its copolymers with other 3-hydroxycarboxylic acids: 3-hydroxyvalerate — PHBV, 4-hydroxybutyrate — P4HB, 3-hydroxyhexanoate — PHHx, etc.). The major physical and chemical and biological properties of PHA, which differ significantly, directly depend on the chemical structure [5, 6]. At that, while chemically synthesized poly(2-hydroxyalkanoates) are widely used to manufacture medical devices, poly(3-hydroxyalkanoates), primarily PHB and its copolymers, are still very limitedly used as medical materials. But the potential of such polymers for regenerative medicine is very high.

High biocompatibility of PHB *in vitro* was demonstrated in studies on cell cultures; the material is recognized as a promising one for tissue engineering. Cell cultures of various types: osteoblast-like cells (MG-63 cells of human osteosarcoma, mouse cells of the MC3T3-E1 line), human, rat, and rabbit MSCs isolated from the bone marrow and the adipose tissue, human and mouse fibroblasts (3T3 line and others), rabbit chondrocytes, human epithelial and endothelial cells, human neurons, rabbit myoblasts demonstrated a satisfactory level of cell adhesion, viability, and proliferation in direct contact with PHB when cultivated on polymer films and scaffolds [13]. Here, the study of the PHB and its copolymers impact on the growth and differentiation of MSCs is particularly important when these biopolymers are used as biomaterials for tissue engineering. Scaffolds from PHB and PHBV ensure a high rate of proliferation of MSCs and osteoblast-like cells [14-19] or, to the contrary, do not support a higher proliferation of MSCs compared to the controls (for example, culture plastic) [20-23], whereas scaffolds from PHHx promote acceleration of cell growth [24-26], but can also cause apoptosis thereof [23]. The PHB high biocompatibility is related not only to the virtual chemical inactivity of the polymer (with the exception of its susceptibility to hydrolytic and enzymatic degradation), but also to the fact that its biodegradation products are not aggressive and toxic, unlike, for example, PLA, which decomposes to quite potent lactic acid, whereas the rate of hydrolytic degradation of PHB is rather low. Generally, PHB biodegradation occurs when it directly destructs phagocytic cells (macrophages, osteoclasts) [13].

It was shown that rat [20, 21, 27, 28], human [29, 30], and rabbit [14] MSCs, mouse osteoblast-like cells of the MC3T3-E1 S14 line [31], human osteoblast-like cells of the MG-63 line [15], and human induced stem cells [16] grown on scaffolds made of PHB and its copolymers PHBV and PHHx are subject to spontaneous differentiation osteogenically under standard conditions (in a standard culture medium), and their differentiation is even more pronounced than with standard stimulation when it occurs under stimulation of osteogenic differentiation (in an osteogenic culture medium). Osteogenic differentiation of MSCs and osteoblast cells grown on scaffolds made of PHB and its copolymers is confirmed by changes in cell morphology [14, 17, 20, 21, 30], suppression of their multiplication [20, 21, 27], increased deposition of calcium salts and alkaline phosphatase activity [15, 19, 20, 28, 32], increased expression of markers of new bone formation and osteogenic differentiation (alkaline phosphatase, osteopontin, osteocalcin, type I collagen, transcription factor 2 — Runx2) [16, 20, 28, 29]; but also there are studies where osteogenic differentiation of MSCs during their cultivation on PHHx scaffolds was not found [24, 25, 33].

It was established [19] that the coating of scaffolds made of the Mg_2_SiO_4_–CuFe_2_O_4_ nanocomposite and a layer of PHB increases cell viability, alkaline phosphatase activity, and scaffold mineralization when osteoblast-like cells are cultivated on it. It was also found [34] that fibrous scaffolds made of the P4HB copolymer by electrospinning significantly facilitate attachment and growth of MSCs isolated from rat bone marrow. The scratch model (mechanical removal of cells on the scaffold surface in the form of a strip of a specified width) also demonstrated that P4HB-fibre scaffolds support MSC migration. Differentiation of MSCs in the osteogenic direction in the osteogenic medium was found, which was clear from an increase in the activity and expression of alkaline phosphatase and in mineralization by scaffold cells. Differentiation of MSCs in the epidermal (into keratinocytes) and endothelial (into vascular endothelial cells) layers was demonstrated in appropriate specific stimulating media that include nanofiber scaffolds obtained by electrospinning and used as a substrate for cell growth [35, 36].

The impact of PHB and its copolymers on the growth and differentiation of MSCs is related to the physical and chemical properties of the polymer material, 3D microstructure, and topography of scaffolds made of this polymer. The treatment of the polymer surface with alkali, enzymes, or plasma, which changes its physical and chemical properties (for example, by increasing hydrophilicity without any microstructure change) leads to a significant increase in cell adhesion and proliferation on the surface of products made of PHB and its copolymers [21, 32, 37–39]. It was assumed that the improved hydrophilicity of polymer films after PHB treatment with lipases, alkali, and plasma allows cells in suspension to easily attach to such films in contrast with the untreated ones. The impact of biomaterial surface hydrophilicity on cell adhesion was demonstrated earlier [40]. Some authors used PHB only as a basis to be covered with biomaterials, the impact of which on the growth and differentiation of MSCs was examined. For instance, type I collagen, with or without chondroitin sulfate in its content, was applied to a scaffold made by easel weaving from PHB fibers, and then the osteogenic differentiation of human MSCs isolated from the bone marrow and growing on the scaffold was assessed [41].

The microstructure and topography of the surface where the cells grow may have a particularly important role in impacting the growth and differentiation of MSCs. Moreover, the impact of microstructure and surface topography can even eliminate the effect of bioactive molecules on cell growth or differentiation [25, 33, 42]. Biological activity of polymers, in particular their impact on the growth and differentiation of MSCs both *in vitro* and *in vivo*, depends on the microstructure of 3D products made of them: surface topography, 3D microstructure, porosity, pore size and shape [3, 5, 25, 33]. Different cells prefer different surfaces: for example, MSCs and osteoblasts prefer rougher surfaces with an appropriate pore size [43, 44], whereas fibroblasts — a smoother surface, and epithelial cells attach only to the smoothest surfaces [45]. Surface roughness affects cell attachment because it provides the required space for the MSCs or osteoblasts to grow, or serves as a strong substrate for attachment of their filopodia.

According to [25, 33], a scaffold with an appropriate pore size provides the best surface properties to attach type II collagen fibers and their penetration into the inner layers of the scaffold seeded with chondrocytes. This could be facilitated by the interaction of extracell matrix proteins with the surface of the material. At that, a study of proliferation and differentiation of the MSCs grown on electrospun scaffolds with randomly and directionally oriented fibers provided contradictory results: directionally oriented fibers stimulated osteogenic differentiation of MSCs [33], or there was no significant difference in cell differentiation compared to the randomly oriented fibers [29]. Moreover, scaffolds with a porous surface can inhibit osteogenic differentiation of the MSCs cultivated in an osteogenic medium [25]. The corresponding surface properties can also facilitate cell attachment and proliferation, providing more space for better gas and nutrient exchange or adsorption of whey proteins [3, 5]. Being biodegradable polymers, natural PHB and its copolymers cause targeted activation of macrophages and osteoclasts, that is the cells that directly contribute to their biodegradation, and the rate of cell biodegradation is directly related to the ability of macrophages and osteoclasts to adhere to the surface of polymers and proliferate on it. Therefore, the surface microstructure has an important role in a study of the cell biodegradation of PHB and its copolymers [46-49].

The authors studied the impact of the surface topography of films made of PHB and its copolymers as a porous structure in the form of honeycombs of different diameters and with different geometric parameters, as well as in the form of columns and grooves [50]. Visualization of the diversity of cell morphology depending on the topography of the substrate was the key purpose of this study. The analysis of the relationship between the shape of the cells and the substrate allowed to compare the features of MSC attachment to the received polymer films from PHB and its PHBV copolymer with a topography made of long longitudinal grooves. In both cases, the cells were elongated in the longitudinal direction: the cells were elongated along the grooves in the structure, being attached by pseudopodia to the adjacent protrusions. The cells attached and growing on the surface of the films of a column topography had a more extended shape than the cells on smooth films without a pronounced surface topography, where they did not have a direction for orienting pseudopodia and attached to holes in the microstructure. MSCs on films with honeycombs topography had different shapes, and their elongation and flattening were related to the pore size. Other studies [25, 33] demonstrate that the availability of pores and their sizes have absolutely different impacts on various cell types. Moreover, different materials have various impacts on cell attachment regardless of the same pore size, and there is no apparent correlation between the pore size and cell behavior. Pore size has a significant impact only on MSC differentiation and tissue formation. The smaller pores (5 μm) of the PLA film, which is similar to PHB in its physical characteristics, provide to form a denser tissue during cartilage formation than larger pores (20 μm). The pores allow the cells to adhere to the film and control the area of tissue formation, whereas cells tend to detach from the flat film surfaces. In this study, we did not observe the impact of various copolymers on the cells behavior; however, the impact of pore sizes on MSC morphology was revealed [50].

The reason for the observed ability of PHB to induce osteogenic differentiation of MSCs and osteoblast-like cells can be related not only to the specific microstructure of scaffolds made of this polymer or the topography of their surface, but also to the natural biological activity of the PHBs biodegradation products: oligomers and 3-hydroxybutyrate (3-HB). It was demonstrated that 3-HB at concentrations ranging from 0.05 to 1.0 mM can both accelerate the proliferation of various cells [51, 52] and stimulate differentiation of MSCs and osteoblast-like cells in the osteogenic direction [53]. The impact of 3-HB on the growth and functional activity of neurons was also found [54, 55]. It should be noted that the major product of PHB biodegradation, 3-HB, is a common metabolite in the body (the so-called ketone body [56]), which has various types of biological activity. Both low-molecular-weight PHB nanoparticles and water-soluble PHB oligomers can impact proliferation and differentiation of MSCs and osteoblast-like cells [51, 52, 57–59].

Copolymerization of PHB with other polymers provides improvement of their mechanical properties and hydrophilicity. For instance, PHB can be chemically modified with polyurethane and 2-aminoethyl methacrylate [60], which results in a clear improvement in the mechanical properties of scaffolds made of them. The PHB chemical structure, in addition to the use of chemical synthesis for copolymerization, can be modified by gamma irradiation, which also allows to change the mechanical and biological properties of PHB for biomedical application [61, 62].

Manufacturing of composites from PHB and its copolymers with other biomaterials is another promising approach to develop scaffolds with improved physicochemical and biological properties [63, 64]. One material does not always show a combination of all the properties required for a medical device operation. Here, technologies for manufacturing scaffolds from mixtures and composites of PHB and its copolymers using various biopolymers and minerals have been actively developed in the recent years [65-67]. The development of particle composites of PHB with mineral biomaterials (hydroxyapatite [68, 69] or bioglass [70-72]) to get scaffolds allows to control mechanical properties, hydrophilicity, and rate of biodegradation of the composite scaffolds by combining the physicochemical and biological properties of each material, and, in turn, to control their interaction with cells. Hydroxyapatite is widely used as a bone substitute due to its biocompatibility and osteoinductivity, which leads to bone regeneration within a short period. This material is chemically and structurally similar to the mineral phase of a native bone [73, 74]. However, hydroxyapatite scaffolds and other products based on this material showed a low elasticity coefficient (increased brittleness), which limits the use of this biomaterial for bone tissue engineering. That is why it cannot be used to perform load-bearing functions in large bone defects, and it is used mainly in the form of granules, grits, and other loose forms [73]. Therefore, the approach to using scaffolds based on PHB composites with hydroxyapatite can be especially promising [64, 74, 75]. For instance, it was demonstrated that the addition of keratin and hydroxyapatite in the form of nanorods to the fibers of a scaffold made of PHB by electrospinning results in a significant increase in their hydrophilicity and mechanical rigidity [76]. Mixing polymers with hydroxyapatite can also be used to develop extended-release medication systems [77].

The most difficult way is the development of complex multidirectional composites of continuous fibers [63], where the reinforcing phase and the matrix are made of materials that are completely different in their physical and chemical properties. This is the most biomimetic structure of composites: sporocarp, wood, endocarp, coral, foam, nacre, skin, cartilage, bone, that is composites consisting of rigid reinforcement impregnated with a hydrogel-like matrix. The composites of such a structure create the most natural environment for cell growth inside them [78].

Polymers and inorganic substances that are used to manufacture PHB composites impact the osteogenic properties of scaffolds made of them. For example, polydopamine coating of P4HB copolymer scaffolds manufactured by rapid 3D prototyping allowed to functionalize scaffolds with growth factors such as BMP-2 [79]. Addition of nanosized hydroxyapatite to scaffolds made of a PHB–keratin composite results in an improvement in the attachment of osteoblast-like cells of the MG-63 line and an increase in the activity of alkaline phosphatase therein [76]. The coating of 3D-printed P4HB scaffolds with polydopamine developed their ability to enhance osteogenic differentiation of MSCs in an osteogenic environment, which was demonstrated by an increase in alkaline phosphatase activity, an increase in calcium salt deposition, and an increase in osteocalcin expression in cells [79].

## Scaffolds based on poly(3-hydroxybutyrate) and its copolymers for bone tissue engineering: studies on critical and non-critical bone defects models *in vivo*

Model studies of scaffolds for bone tissue engineering provide assessment of the material properties in the long term taking into account various operating factors. Here, it is possible to assess tissues not only in the immediate vicinity of the device, but also in the remote body areas, which is especially important for studies of fragments of matrices, particles, etc. For example, residual elements in a human body can be transferred to organs such as the liver and spleen [80]. However, it should be remembered that such models are only an approximated picture of how various products will actually behave in the body. At that, each model has its own advantages and disadvantages. At present, there are many models for testing implant materials *in vivo*, ranging from assessment of protein adsorption of blood components to studies of bone integration and implant degradation.

There is an enacted regulatory document — GOST ISO 10993-6—2011 — to regulate studies of the medical materials and products in the Russian Federation. For instance, it describes the methods of subcutaneous, intramuscular and intraosseous implantation of polymeric biodegradable products into the body of rats, rabbits, pigs, etc. It is proposed to use various histological parameters, such as inflammation, tissue reaction, fibrosis, necrosis, and etc. to assess biocompatibility [81].

In order to test scaffolds for orthopedics or maxillofacial surgery, the majority of authors create artificial bone defects. There are two types of models of such defects in studies of the regenerative potential of scaffolds based on PHB and its copolymers, their composites, and other biomaterials: non-critical bone defects (for example, 1–3 mm in diameter in rats) that can heal themselves, and critical bone defects (for example, with a diameter or linear dimensions of more than 6 mm in rats), which cannot heal themselves. Such defects are usually made to the femurs and bones of the cranial vault in laboratory rodents (rats and mice), after which bone tissue regeneration is examined by using computed tomography, histology, and immunohistochemistry.

**Figure 1.**
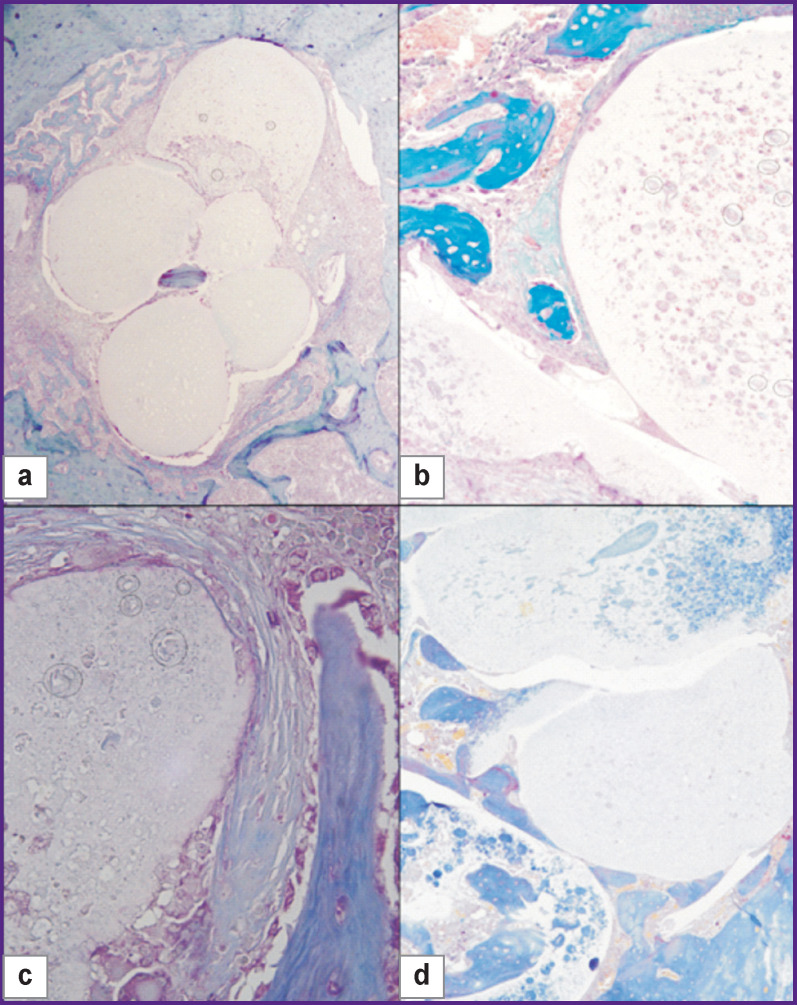
Histological section of the matrix samples from large microspheres in rat femur (Mallory stain): (a) in 6 days; (b) in 14 days; (c) in 30 days, and (d) in 90 days after surgery [27]

The osteoinductive properties of PHB and its copolymers are manifested not only *in vitro*, but also *in vivo*, when scaffolds made of these polymers were used as bone-replacing biomaterials and were implanted into the area of critical and non-critical bone defects. It was demonstrated [13, 20, 82, 83] that PHB scaffolds stimulate bone tissue regeneration and formation of new bone tissue (found by histology and immunohistochemistry by the expression of bone tissue development markers, such as osteopontin and type I collagen). The authors have previously shown on a noncritical bone tissue defect [27, 84, 85] that porous microspheres from PHB stimulate germination of vessels in the pores of the microspheres and formation of a new reticulo-fibrous bone tissue in the pores (on day 6), the small sites of which begin to merge later (day 30), and eventually (in 100 days), the newly formed lamellar bone completely fills the microspheres, which is accompanied by active resorption and replacement of the polymer material. Here, we did not observe formation of a fibrous capsule around the PHB implant, which indicates the complete integration of the biomaterial with the bone tissue (Figure 1).

Other authors have also shown that PHB and its copolymers promote bone tissue regeneration in a non-critical bone defect model. For instance, it was demonstrated that the implantation of scaffolds from the Mg_2_SiO_4_-CuFe_2_O_4_ nanocomposite coated with PHB into a rat femur for 8 weeks resulted in an improvement in bone tissue regeneration compared to the control group, as evidenced by the results of microcomputed tomography, including analysis of the volume fraction of bone tissue and thickness of trabeculae [19].

**Figure 2.**
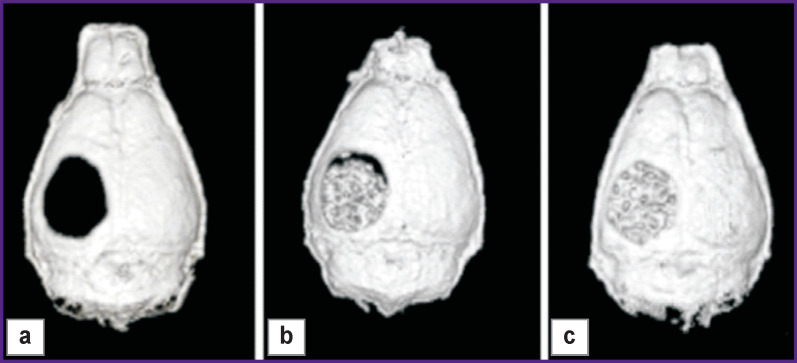
Critical defect in the parietal bone of a mouse 8 weeks after implantation of the scaffold [83]: (a) without a scaffold; (b) with a scaffold; (c) with a scaffold seeded with mesenchymal stem cells

Many authors [82-84] successfully restore bone defects using, for instance, MSCs isolated from the bone marrow, as well as stromal cells from the adipose tissue. For example, non-healing, critical-sized (4 mm) defects of the calvarium were drilled in the right parietal bone of adult nude mice of the BALB/c line [83]. A porous ceramic scaffold based on a composite of 60% hydroxyapatite and 40% calcium β-orthophosphate was placed in the defect area immediately after its drillage. In the control group, bone defects remained uncovered being covered with skin over time. During 8 weeks, the mice were examined using microcomputed tomography methods and taken out from the experiment at the end of this period. Scaffolds were pre-seeded with human MSCs. A no-cell scaffold was introduced in some animals as an additional control. The defect types are shown in Figure 2. The results of this study showed that MSCs on scaffolds are appropriate for treatment of such cranial defects in mice.

Another group of scientists [82] conducted similar studies, but the matrix there was loaded with a chemoattractant, a stromal cell factor (SDF-1α). This protein made MSCs move along its concentration gradient, which also resulted in restoration of a skull defect of a critical size.

It should be noted that the Russian state standard (GOST) does not recommend studies on the bone tissue of small rodents, such as rats and mice, as the minimum defect size prescribed there (2 mm) can hardly be made on small bones [81]. However, taking into account the convenience of working on rodents, such studies are still performed with the largest bones selected. That is why the models on the skull of rats and mice are that often. Here, rats are more suitable as their bones are meaningfully larger.

Before providing examples of studies involving scaffolds made of PHB and its copolymers on bone defects, one must mention an interesting work by Rentsch et al. [85]. The scientists performed subcutaneous implantation of a machine-spun PHB scaffold that was coated with collagen and/or chondroitin sulfate and seeded with MSCs to athymic rats. Six weeks after the implantation, the authors found that active vascularization occurred in the connective tissue capsule around the scaffold and the expression of the following osteogenic markers was increased: type I collagen, osteopontin, osteonectin, osteocalcin, and bone sialoprotein; at that, no heterotopic ossification was seen. Another study by German scientists [86], using the same scaffold based on PHB demonstrated a more pronounced ectopic formation of bone tissue in the case of using a scaffold based on PHB; here, the scaffold was seeded with primary osteoblasts isolated from the jaw bone tissue, and compared with a scaffold made of a hydroxyapatite-collagen composite, and implanted intramuscularly to athymic nude rats.

**Figure 3.**
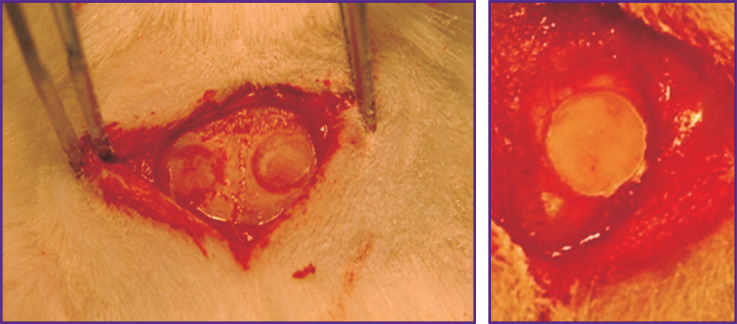
Two bone defects made next to each other in one animal, and a defect covered with a poly(3-hydroxybutyrate) plate [87]

The most indicant study on a critical bone defect is the study conducted by Gredes et al. [87]. The scientists made two defects located side by side to the skull of a rat. Due to this, a greater experiment purity was achieved, as the experimental, i.e. the defect repaired with the matrix, and the control (without implantation) defects were in absolutely equal conditions in the same animal (Figure 3). This was done with a trepan while constantly washing the wound surface. Here, the PHB plate demonstrated good ability to restore the bone tissue. In 12 weeks after implantation, bone defects were healed by 54%, which was accompanied by a pronounced vascularization and a significantly increased expression of osteogenic markers compared to the control: type I alkaline phosphatase, type I collagen alpha-1, and vascular endothelial growth factor A, which indicates an expressed osteoinductive effect.

In another study [34], scientists used a bilateral critical bone defect of the parietal bone. The diameter of each defect was 5 mm; one of the defects was covered with a fibrous scaffold made of the P4HB copolymer. 12 weeks after the surgery, many new bone formations were visually registered. The concentration of striated collagen bundles during week 4 and newly formed bone tissue in the area of the defect on week 12 with the implanted P4HB scaffold was registered compared with the control group where no scaffold was applied. Scattered collagen bundles started from the edges of the defects and stretched into scaffolds, growing along their polymer fibers. Moreover, denser collagen bundles in the form of reticulo-fibrous bone tissue were seen near the edge of the defect, which indicates that electrospun P4HB scaffolds can facilitate bone tissue maturation.

A study on a single critical bone defect of the rat parietal bone with a diameter of 5 mm [79] demonstrated that 3D-printed scaffolds made of P4HB and coated with polydopamine with the specifically adhered human recombinant growth factor BMP-2 had an express ability to accelerate bone tissue regeneration; this was less true for scaffolds made of P4HB and uncoated or coated with polydopamine, and true to a greater extent for BMP-2 scaffolds. In their study, Higuchi et al. [88] made a similar defect in the lower jaw of a rat, and then introduced a scaffold of a PLGA composite and collagen loaded with BMP-2. After that, the muscle fibers and skin were sutured. 2 weeks later, a study of osteogenesis on rats withdrawn from the experiment was performed. It was demonstrated that in 4 weeks the group with a scaffold without growth factor had minimal development of newly formed bone tissue, whereas in case of a scaffold loaded with BMP-2, bone tissue regeneration was more active, which evidences the absence of osteoinductive properties in synthetic PLGA.

**Figure 4.**
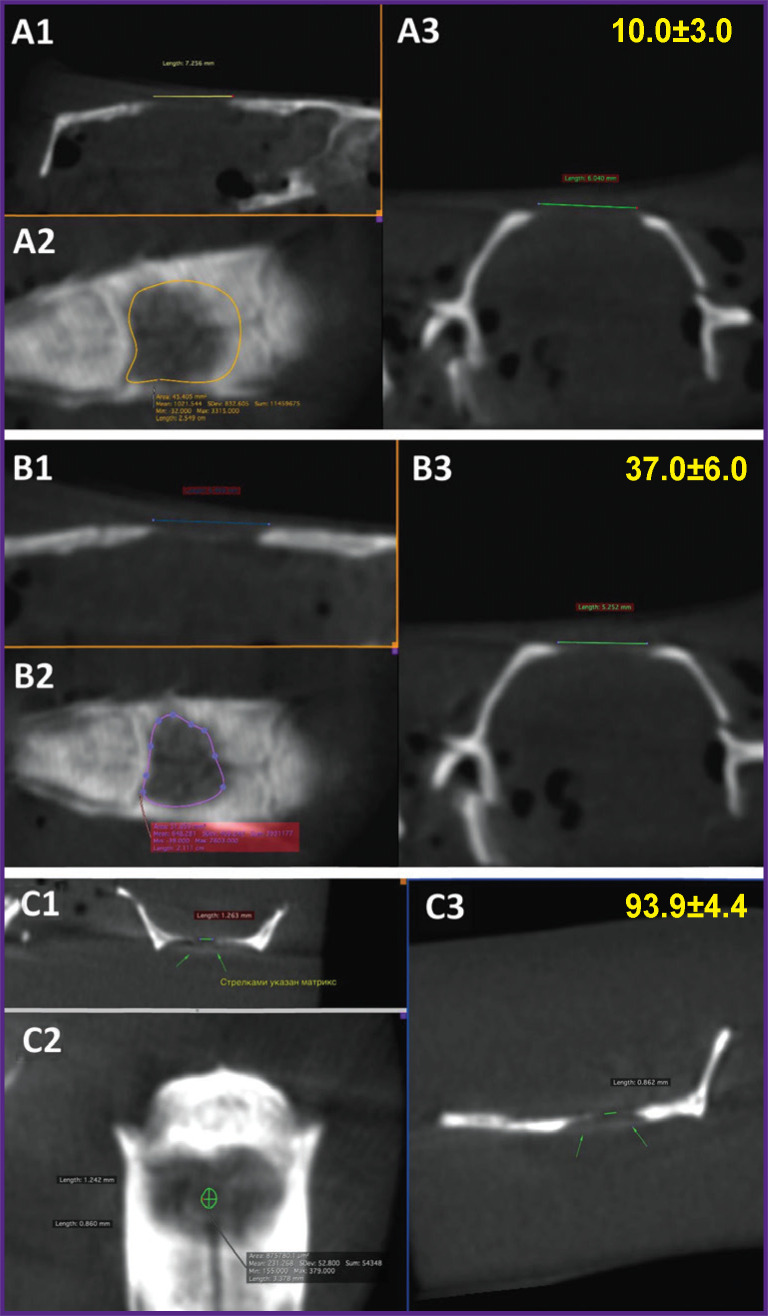
Computed tomography (sagittal (1), axial (2), and coronal (3) projections) of a critical bone defect in a rat parietal bone 28 days after implantation of the cell-free (b) and seeded (c) scaffolds compared to the control bone defect without scaffold implantation (a) Numbers in yellow in the upper right corner indicate the percentage of defect closure in each group [27]

Using a single critical defect of the rat parietal bone of 8 mm in diameter with a PHB scaffold filled with alginate hydrogel, we have previously [27] shown that 28 days after implantation aimed to close the defect area it could induce active regeneration of bone tissue with a 37% defect recovery, whereas the scaffold seeded with MSCs resulted in the restoration of a critical bone defect by 94% (Figure 4).

**Figure 5.**
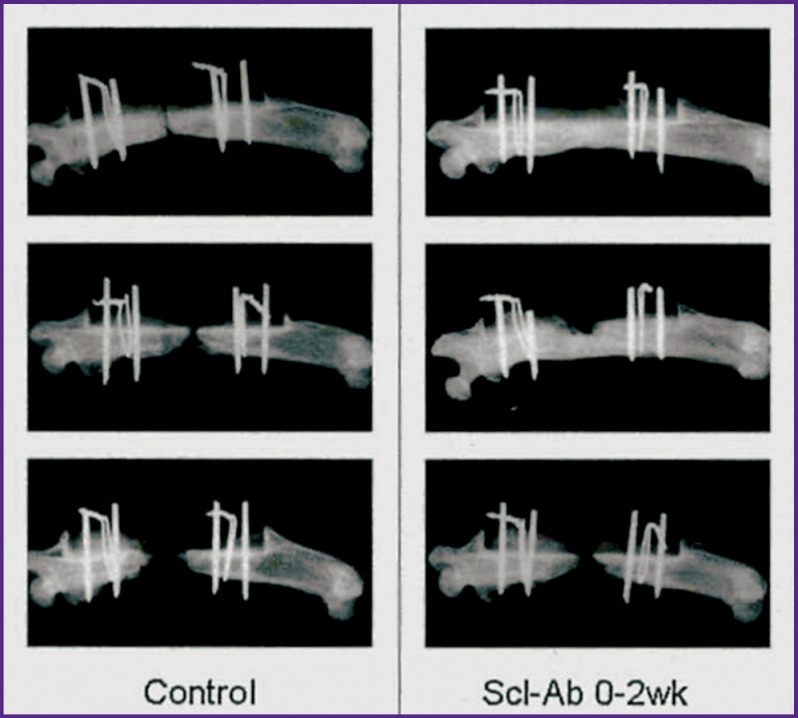
Radiographs of the defect site in the rat femur after administration of antibodies blocking sclerostin [89]

Other defect models are used in reconstruction of skeletal components involved in the musculoskeletal system, such as, for example, the femur. It should be noted that the authors are again primarily interested in rodent models due to the simplicity of working with these animals, as well as the affordability of such experiments. Virk et al. [89] used a widely accepted approach in their study. They excised a critically sized shaft of a rat femoral segment following the protocols of the Institutional Animal Care and Use Committee. Here, the critical is a size of the defect which can not correctly heal itself without orthopedic assistance. This study is of interest as it does not involve a scaffold matrix but systemically administers antibodies that block sclerostin, an antagonist protein that regulates the osteoblast spread during the bone formation. The bone in this case is fixed with frame elements. Figure 5 visually demonstrates the impact of antibodies on osteogenesis and defect healing. The bone pieces grow back together better than in the control.

**Figure 6.**
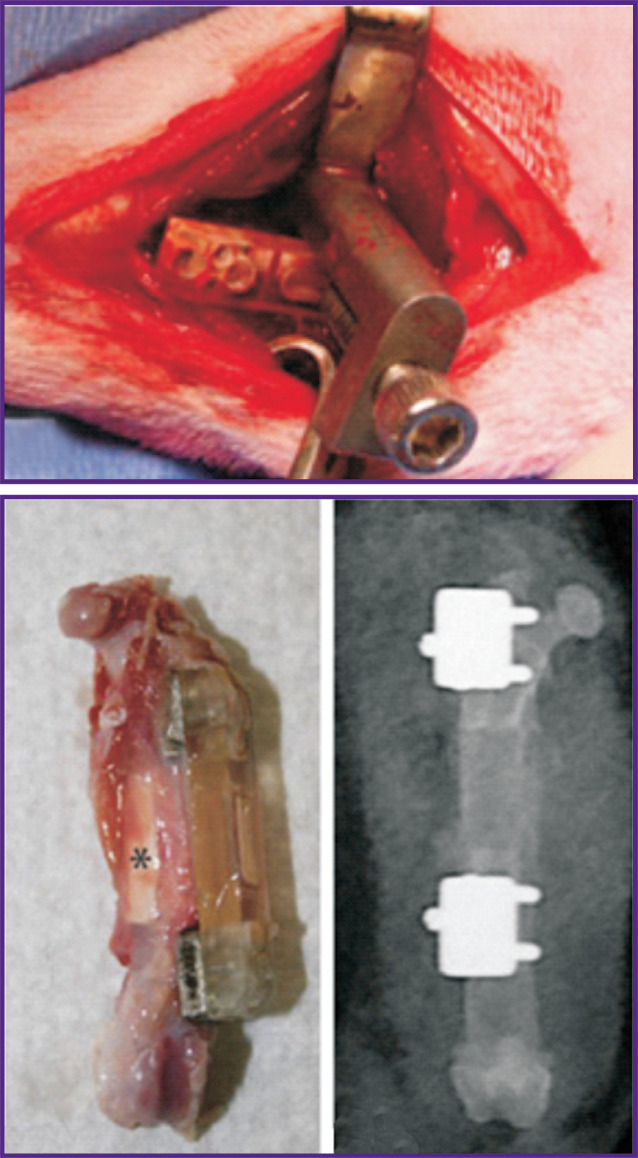
Introduction into the body and general view of a bone implant [90] * indicates nanofiber mesh tube

Such studies are more wide-spread among those who adhere to the use of biomaterials and the corresponding orthopedic implants. For instance, Berner et al. [90] applied this model to test a mineralized tube made of an electrospun polycaprolactone fabric. A defect which was made in a similar way (Figure 6) [90] was additionally fixed with iron reinforcement. A decent replacement of the matrix with bone tissue is performed with the support of an osteogenesis agonist — BMP-7 protein. The experiment was also precisely monitored using micro-CT and radiography. Moreover, this group performed a biomechanical study of the newly formed bone *ex vivo*.

A large number of scientists (e.g., Li et al. [91] and others) conducted the experiment similarly due to the logic of this model application to test various approaches to osteoneogenesis, although the ways of solving this problem are diverse.

**Figure 7.**
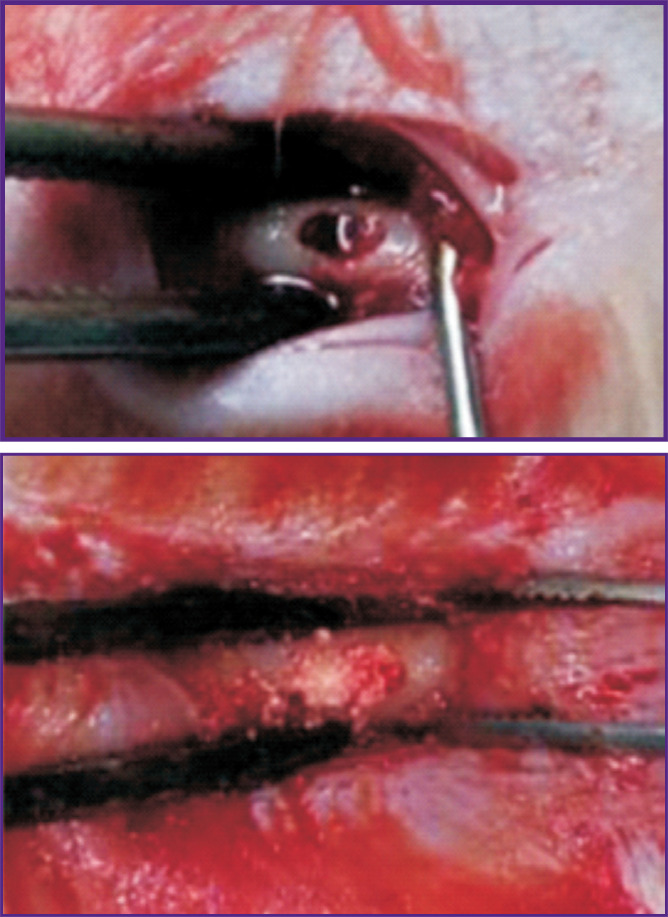
Local defect of the rat femur [92]: before and after filling with stem cells with an agent inducing osteogenic differentiation

It should be noted that the considered models of critical defects are used at later stages of pre-clinical studies, when the osteoplastic material has already proven its biosafety and the scientists are interested in its therapeutic effectiveness. When a scientist faces not a quantitative (how long and how effectively the defect will be restored), but a qualitative question, that is whether the bone cells are formed at all, then a small hole in the femur is often sufficient. For example, Feng et al. [92] used histochemical methods to study osteogenic differentiation of the stem cells isolated from the adipose tissue at the site of a drilled bone defect of 3×5×5 mm in size (Figure 7).

## Conclusion

The osteoinductive properties of poly(3-hydroxybutyrate) and its biodegradation products make this polymer a promising material for bone tissue engineering. Scaffolds and microspheres based on poly(3-hydroxybutyrate) and its copolymers can be used to develop an osteoplastic material for the bone defects restoration in maxillofacial surgery and orthopedics; here, this material will have both osteoconductive and osteoinductive properties. Moreover, they can serve as a platform for development of several products with additional bioactivity types due to filling with various agents: low-molecular-weight medications, peptides, growth factors, exosomes, and stem cells.
